# Autism Spectrum Disorder in Children Adopted After Early Care Breakdown

**DOI:** 10.1007/s10803-015-2680-6

**Published:** 2016-01-06

**Authors:** Jonathan Green, Kathy Leadbitter, Catherine Kay, Kishan Sharma

**Affiliations:** Social Development Research Group, Institute of Brain Behaviour and Mental Health, University of Manchester, Room 3.311, Jean McFarlane Building, Oxford Road, Manchester, M13 9PL UK; Pennine Care NHS Foundation Trust, 225 Old St, Ashton-under-Lyne, Manchester, Lancashire OL6 7SR UK

**Keywords:** Autism spectrum disorder, Adoption, Neglect, Maltreatment, Early adversity, Pre-natal adversity

## Abstract

Syndromic autism has been described in children adopted after orphanage rearing. We investigated whether the same existed in children adopted after family breakdown. Families of 54/60 adopted children aged 6–11 years (mean 102 months; SD 20; 45 % male) returned screening questionnaires for autism spectrum disorder (ASD); 21/54 (39 %) screened positive. Detailed in-person phenotyping of screen positive cases showed ASD in 6/54 (11 %), Broad ASD (sub threshold traits) in 10/54 (18.5 %); 5/54 (9 %) screened false positive. The ASD group showed impairments across both social communication and restrictive repetitive behaviour domains, Broad ASD was more mixed. These rates, much higher than population prevalence, are comparable with institutionalised samples. There are implications for developmental science, and assessment, treatment and policy for adopted children.

**Electronic supplementary material:**

The online version of this article (doi:10.1007/s10803-015-2680-6) contains supplementary material, which is available to authorized users.

## Introduction

Findings from longitudinal studies of children adopted internationally following severe early global deprivation in orphanages have demonstrated a significant impact of adverse environmental experience on later social development, including a pattern of ‘quasi-autism’ (Q–A), found for instance in 6.3 % of 165 children at 6 years in the England-Romanian Adoptees (ERA) study, with a similar additional number of children displaying partial features (Rutter et al. 1999). Key features of the described syndrome were difficulties in forming selective friendships, impaired social reciprocity, eye contact, social gesture and a lack of reciprocal social exchange, sensory preoccupations and intense circumscribed interests (as opposed to the stereotyped behaviours such as rocking previously associated with institutional rearing). The profile differed from ‘classical’ autism in an increased amount of social approach and spontaneous communication, equal sex ratio, non-elevated head circumference and (as it proved on further follow-up) a developmental course rather unlike classical autism. Thus in ERA at 11 years the Q–A pattern persisted in 75 % of children, but in others had become modified to resemble the unusual social behaviour known as Disinhibited Attachment Disorder (DAD), observed separately in 39.1 % of the cohort at 11 years (Rutter et al. 2007; Kumsta et al. 2010). At 18–20 years, 15 young people still met criteria for Q–A (compared to 16 at 6 years), the majority of these cases still showed Q–A characteristics such as unusual obsessional behaviours and circumscribed interests (Kreppner et al. 2010), but there had often been a lessening in social impairment, and frequent overlap with criteria for DAD (Kumsta et al. 2010). Severe early institutional deprivation was thus associated with a striking autism-like syndrome in early childhood in up to 13 % of the sample - substantially higher than would be expected from population-based prevalence rates for ASD, which are consistently around 1 % across global studies (Elsabbagh et al. 2012). The developmental course of this Q–A tends to modify towards the broader autism phenotype, although core social impairments usually persist; presence of Q–A in adolescence is associated with raised rates of other psychopathology, functional impairment and increased service use (Kumsta et al. 2010; Kreppner et al. 2010).

This identification of Q–A was in a very particular population group and a key question follows as to whether similar impairments might also occur in children who had been adopted after severe early social neglect or maltreatment within families (since high income societies have largely abolished early institutional care). A national survey of 1253 children aged 5–17 years in local authority care in Britain found a prevalence of caregiver-reported autism-like symptoms of 2.6 %, but there was no in-person phenotypic ascertainment (Ford et al. 2007). ‘Autistic-like detachment’ was described in 26 % of 70 children at care-entry between 5 and 12 years (Dimigen et al. 1999) but this had not been assessed in relation to defined Q–A or ASD. More recently, 46 % of 35 children (5–8 years) selected for Reactive Attachment Disorder (RAD) after early maltreatment showed autism symptoms on standard caregiver interview (Sadiq et al. 2012). Such studies suggest the possibility of increased rates of ASD in high-risk children in out-of-home care, but lack direct and detailed phenotypic ascertainment. Further, they beg the aetiological question of the origin of any ASD found. In the ERA study, a primary effect of the institutional environment was supported by a dose–response relationship between length of post-natal institutional exposure and Q–A outcomes, along with available data suggesting that prior familial and prenatal biological risks were unlikely to account for the symptoms (Kumsta et al. 2010). However, in the context of familial neglect and maltreatment equivalent assumptions cannot be made: as well as the potential direct effect of severe neglect or maltreatment itself there may be a convergence of other pre-, peri-, and post-natal risks associated with a maltreating environment, such as familial genetic risk, prenatal exposure to alcohol, drugs, infection, prematurity, poor nutrition, or major psychological stress, all themselves recently linked with potential neurodevelopmental impacts (Miles et al. 2003; Ronald et al. 2011; Rai et al. 2013; Bromley et al. 2013; O’Connor et al. 2014).

Against this background, we designed to our knowledge the first systematic ascertainment of possible ASD in children adopted after early care breakdown, a group associated with severe early neglect or maltreatment (Rees and Selwyn 2009) but likely also to have suffered other pre-natal risk exposure. Phenotypic ascertainment used a two-phase design, with initial standardised screen followed by in-person phenotypic ascertainment using gold standard assessment instruments for ASD. The study was also designed to address as completely as possible, potential familial, prenatal or postnatal risk exposures that might be associated with any ASD identified.

## Methods

### Sample

Sixty children aged between 6 and 11 years old, adopted from UK care, were recruited from national web and mailshot advertisement by Adoption UK, a national membership charity for adoptive families. The study was advertised as a ‘study of social outcomes after adoption’, with neither autism nor any specific hypotheses mentioned. Families opted in by contacting the research team. Sample characteristics are described below.

### Measures

#### Maltreatment and Care History

Data on maltreatment and care history was collected from adoptive parent report, including age at entry into care, number of care placements, age at adoption and experience of maltreatment. For the sub-sample identified after positive phase 1 screen (see below), more detailed family and developmental history was obtained from structured caregiver interview, covering developmental and mental health history in the birth family, prenatal and perinatal risk exposure, and known details of postnatal care.

#### Psychopathology (Whole Sample)

The online Development and Wellbeing Assessment (DAWBA) was used to assess psychopathology. The DAWBA is an extensively validated web-based parent-report questionnaire consisting a series of fixed choice response (i.e. does your child worry?) and open ended (i.e. what does your child worry about?) questions regarding behaviour and symptoms associated with DSM-IV diagnostic criteria for a range of emotional, behavioural, developmental (including ASD) and hyperactivity disorders. A computer program uses captured information to predict likelihood, within six probability bands ranging from <.1 % to >70 % chance, of meeting DSM-IV criteria for each diagnostic category. A trained clinical rater reviews all evidence, including open-ended responses to accept or override the computer-generated diagnoses. The clinical reviewer’s decision ratings (present [1] or absent [0]) for each diagnostic category were used in analysis. The DAWBA has been used in a number of large-scale epidemiological studies to assess mental health needs of children in the UK (Meltzer et al. 2000; Ford et al. 2007) and the diagnostic algorithms have been developed based on analysis and independent clinical review of over 20,000 cases in international survey studies. Studies show moderate to high agreement (Cohen’s kappa of .63–.94) between diagnostic classifications from clinical review of DAWBA and independent clinician diagnoses (Alyahri et al. 2006; Aebi et al. 2012; Colvert et al. 2015).

#### ASD Screen and Phenotyping

Whole sample phase 1 screen for ASD phenotype used the DAWBA ASD module, which contains a mixture of fixed choice and open-response items structured around DSM-IV and ICD-10 criteria for ASD (Ford et al. 2007; Colvert et al. 2015). In the current study, children were identified as ‘screen positive’ if they showed: (1) ≥15 % predicted risk on the ASD module of DAWBA; or (2) ≥5 % predicted risk for ASD plus positive clinical judgement on independent clinical review of open and fixed choice respondent information; or (3) reported prior independent ASD diagnosis.

*Phase 2 phenotyping* for screen positive cases used (1) Autism Diagnostic Interview-Revised (ADI-R); (Le Couteur et al. 2003) a standardised, investigator-based developmental interview used in the diagnosis of ASD, scored within domains of early childhood and current communication, social development and restricted, repetitive, stereotyped behaviours and interests, based on behavioural descriptions at 4–5 years for some items and at any time in development for others; and with established cut-off for childhood autism. (2) Autism Diagnostic Observation Schedule-2 (ADOS-2); (Lord et al. 2012a, b) a semi-structured, assessor administered, play-based assessment, videotaped for independent coding, focusing on the domains of reciprocal social interaction, language and communication, imagination and stereotyped behaviours and restricted interests in four modules appropriate to the child’s expressive language skills and chronological age, with algorithm and autism and ASD cut-off scores in domains of Social Affect and Restricted and Repetitive Behaviour.

#### Language and Communication

*Verbal competency* was assessed using the Word Classes and Sentence Recall subtests of the Clinical Evaluation of Language Fundamentals-4 (CELF-4), used as validated indicators of expressive and receptive language skills; highly correlated with expressive (r = .81) and receptive (r = .85) language scores (Botting and Conti-Ramsden 2008). *Pragmatic language skills* was assessed on the Children’s Communication Checklist-2 (CCC-2; Bishop 2001); a validated 70 item parent-rated questionnaire indicating the frequency of communicative problems grouped in 10 subscales, with a General Communication Composite and a derived Social Interaction Deviance Composite identifying children with pragmatic difficulties disproportionate to structural language.

#### Physical Measures

In order to index the biological impact of risk exposures, we included standard measures of head circumference and examination for 30 minor physical anomalies (MPA’s) adapted from Jenkins (Jenkins 2006). Extremes of head circumference and high numbers of MPAs have been associated with neurodevelopmental disorder. Presence of Foetal Alcohol Spectrum Disorder (FASD) was recorded if full standard Hoyme criteria were met (Hoyme et al. 2005), including a history of alcohol exposure in pregnancy, characteristic facial abnormalities, growth retardation and evidence of complex behavioural and cognitive difficulties.

### Procedure

Data on demographics, care history and post-natal adversity including maltreatment and neglect was collected from the whole sample. All parents were invited to complete the DAWBA screen online with telephone support as needed. Independent clinical evaluation within the DAWBA was blind to detailed study hypotheses. Children screening positive were then invited to complete the phase 2 in depth assessment including definitive phenotyping using ADOS, ADI-R, biometrics, language assessment and developmental interview during a home visit. The assessor for the phase 2 ascertainment was a highly experienced autism researcher, independent of the core study team and with extensive experience of assessing children with idiopathic ASD on these instruments. All ASD cases and a proportion of Broad ASD cases were subsequently seen additionally by the first author for separate clinical evaluation (with the exception of 2 autism cases who had already had full autism clinical assessment by local services). The study was approved by the University of Manchester Research Ethics Committee. Fully informed written consent was obtained from a parent or legal guardian prior to participation.

### Analysis

**Fig. 1:**
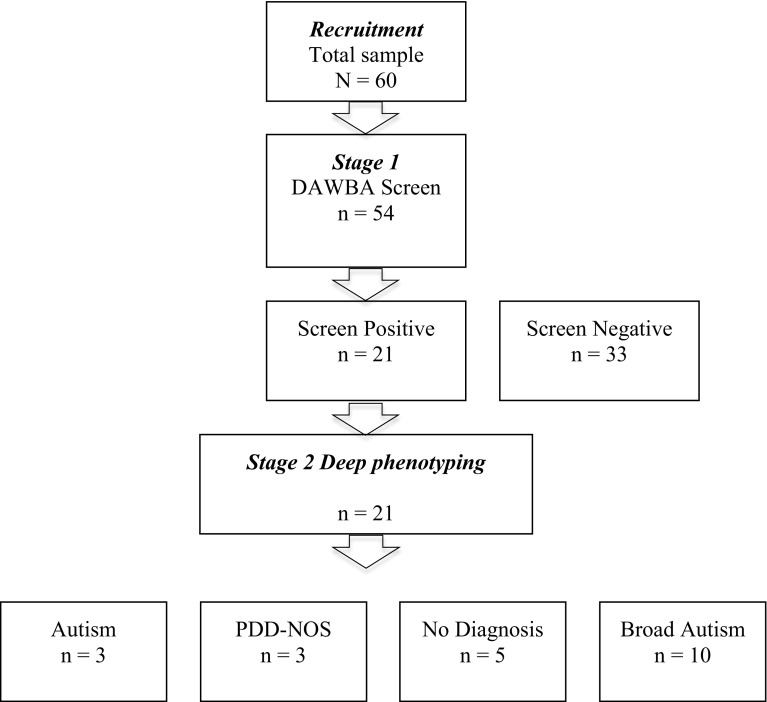
Flow chart of study

Diagnostic classification of ASD used the algorithm developed by the National Institute of Child Health & Human Development (NICHD) Collaborative Programs of Excellence in Autism (CPEA; Lainhart et al. 2006); developed to standardise clinical diagnostic practice using ADI-R and ADOS across specialist centers and used in a large multisite confirmatory study of autism diagnosis (Lord et al. 2012a, b). In the CPEA algorithm, a classification of ‘autism’ meets criteria for autism on all domains of ADI-R and ASD cutoff criteria on ADOS; ‘PDD-NOS’ meets criteria for autism on either social or communication domain of ADI-R, with the other within two points of cutoff, plus meeting ASD criteria on ADOS. Classification of ‘Broad ASD’ is given to subjects with a sub-diagnostic threshold partial phenotype, although the CPEA consortium consider that this group will include “many individuals who may have met criteria for autism, Asperger syndrome, or PDD-NOS if additional data were available” (Lainhart et al. 2006 p. 2259). Classifications are independent of IQ criteria if the mental age is >18 months, something that applied to all study children, indexed by report and measured verbal ability. Within DSM5, individuals within these ‘autism’ and ‘PDD-NOS’ categories are now classified as ASD, provided both social communication and RRB criteria are met; for the purposes of this paper we therefore describe this combined group as ASD and distinguish ‘autism’ and ‘PDD-NOS’ categories within that. Applying DSM5 criteria, one child in our sample, classified by CPEA criteria as PDDNOS, would be re-assigned to ‘Broad ASD’. To avoid any confusion in relation to DSM5, our results reported here therefore reflect this re-assignment (see Table 2). Distribution of demographic, language and other psychopathology variables across classification groups are assessed using ANOVA and Chi-2 statistics (Fig. 1).

## Results

### Sample Characteristics

At assessment the total sample (*n* = 60) had a mean age of 102 months (*SD* = 20) and included 27 (45 %) boys and 33 (55 %) girls. Fifty-five (93 %) of the adoptive parents had a higher degree or professional qualification. Mean age at entry to local authority care had been 12.5 months (*SD* = 15.6, range 0 (birth) to 60). Mean number of care placements was 3.1 (SD = 1.5; range 2 to 10) and mean length of time spent in out-of-home care prior to adoption was 24.3 months (*SD* = 22.2, range 1 to 132). Mean age at adoption was 35.5 months (*SD* = 27, range 3 to 132). All children entered care due to child protection concerns about actual or potential harm and were adopted from foster care. Seventy two percent (43/60) of the total sample had been exposed to maltreatment and 53 % (32/60) had experienced ≥ two forms of maltreatment. Fifty five percent had experienced pre-natal adversity i.e. exposure to pre-natal maternal drug or alcohol misuse (18/60 [30 %] suspected exposure, 6/60 [10 %] documented exposure, 9/60 [15 %] with physical symptoms at birth that required treatment). Six (10 %) had no known experience of either pre- or post-natal exposure; 22/60 (37 %) had both pre and post-natal exposure; 21/60 (35 %) experienced just post-natal and 11/60 (18 %) had just pre-natal exposure.

### ASD Ascertainment

Ninety percent (54/60) of participants completed the DAWBA screen. DAWBA respondents showed no significant difference in age (*U* = 154, *p* = .84), gender (χ^2^ = .67, *df* = 1, *p* = .57) or ethnicity (χ^2^ = 1.3, *df* = 1, *p* = .259) compared to non-respondents. Twenty-one (39 %) of these 54 respondents met DAWBA screen criteria for ASD (see methods). Detailed phenotypic assessment was completed on all these 21 screen positive children and detailed results are in Table 1 with summary demographic, language and psychopathology variables across groups in Table 2. CPEA criteria for ASD (‘Autism’ or ‘PDD-NOS’) were met in 11 % (6/54) and for ‘Broad ASD’ in a further 18.5 % (10/54); 9 % (5/54) were false screen positives.

**Table 1:** Details of children within CPEA classifications

Participant Number	Gender	Age (y;m)	Known birth history of ASD?	ADI-R cut-off met			ADOS-2				
				Social interaction	Communication	RRSPB^a^	Module	Total score	Meets ASD cut-off?	Evidence of RRB^b^	CPEA^c^ category
001	M	7;04	Y	Y	Y	Y	2	13	Y	Y	Autism
002	F	7;11	Y	Y	Y	Y	2	23	Y	Y	Autism
003	M	12;09	N	Y	Y	Y	3	19	Y	Y	Autism
004	M	10;02	N	Y	Y	N	3	7	Y	Y	PDD-NOS
005	F	8;07	N	N (−1)^e^	Y	Y	3	10	Y	Y	PDD-NOS
006^d^	M	11;09	N	Y	Y	N	3	8	Y	N	PDD-NOS
007	F	8;06	N	Y	Y	N	3	8	Y	Y	PDD-NOS
008	M	9;02	N	Y	N	Y	3	5	N	Y	Broad ASD
009	M	12;05	N	Y	Y	Y	3	5	N	N	Broad ASD
010	F	12;06	N	Y	N	Y	3	1	N	N	Broad ASD
011	M	12;02	N	Y	Y	N	3	3	N	Y	Broad ASD
012	M	9;09	N	Y	Y	Y	3	3	N	N	Broad ASD
013	M	7;05	N	N (−1)^e^	N (−1)^e^	N	3	9	Y	N	Broad ASD
014	M	9;09	N	Y	N	N	3	8	Y	Y	Broad ASD
015	M	9;11	N	Y	Y	Y	3	1	N	N	Broad ASD
016	F	6;08	N	Y	Y	Y	3	3	N	N	Broad ASD

^a^Restricted, repetitive and stereotyped patterns of behaviour

^b^Restricted and repetitive behaviours

^c^Collaborative Programmes of Excellence in Autism

^d^This case showed an absence of ADOS RRB and in the light of later DSM5 criteria has been re-assigned for this report into the Broad ASD category

^e^Missed cut-off by one point, allowing categorisation within PDD-NOS within CPEA criteria (see text)

**Table 2:** Demographic, language and psychopathology variables across ASD groups

Variable		Group					Statistics		
		Autism	PDD-NOS	Broad autism	No diagnosis	Screened negative	*K*–*W*	*t*	Sig.
*Demographics*									
Age	Median months	84	99	108.5	93	97	2.44	4	.655
							*X* ^2^	*df*	Sig.
Gender (male)		2 (67)	1 (33)	8 (80)	2 (40)	12 (35)	7.01	4	.135
Ethnicity (White British)	N (%)	3 (100)	2 (67)	10 (100)	5 (100)	27 (79)	4.91	4	.296
Learning difficulties (parent report)		3 (100)	1 (25)	2 (22)	0	1 (3)	25.86	4	.000
SEN schooling		2 (66)	0	0	0	0	35.98	4	.000

							*K*–*W*	*df*	Sig.
*Language*									
CELF word classes^a^	Median (N)	23 (1)	28 (2)	22(8)	30 (5)	27 (32)	1.02	4	Ns
CELF sentence recall		47 (1)	44 (2)	51 (8)	33 (5)	49 (32)	5.39	4	Ns
CCC2 General Communication Composite^b^	Median Scaled Score (N)	3 (2)	3 (2)	1 (9)	3 (5)	43 (34)	21.86	4	.000
CCC2 social interaction deviance composite		−10	−13	−4	−1	−2	4.48	4	Ns
CCC2 syntax		4	26	6	7	11	12.89	4	.12
CCC2 semantic		3	22	4	4	10	14.15	4	.007
CCC2 coherence		4	2	1	6	9	21.36	4	.000
CCC2 inappropriate initiation		4	1	1	5	7	15.78	4	.003
CCC2 stereotyped		3	9	3	5	10	17.69	4	.001
CCC2 context		1	2	0	4	9	21.03	4	.000
CCC2 nonverbal		1	1	1	5	8	24.68	4	.000
CCC2 social		0	1	0	3	7	23.59	4	.000
CCC2 interests		4	3	1	5	8	17.10	4	.002

							*X* ^2^	*df*	Sig.
*Psychopathology* ^c^									
Emotional disorder	N (%)	1 (33)	3 (100)	5 (56)	2 (40)	6 (17)	12.14	4	.016
ADHD		2 (66)	2 (67)	7 (78)	4 (80)	9 (26)	12.26	4	.016
ODD/CD		2 (66)	1 (33)	7 (78)	2 (40)	13 (38)	5.26	4	.262
DAD		0	2 (67)	7 (70)	3 (60)	15 (44)	4.60	4	.330

^a^Median score (N), higher score equates to better functioning

^b^Median Scaled Score (N), higher score equates to better functioning

^c^Psychopathology data missing for 1 participant in the Broad ASD Group

The three children (5.6 %) meeting ‘*autism*’ *criteria* (two boys, one girl) all showed classical autism phenomenology with high ADOS-2 total scores well above the core autism threshold. They showed significant impairments in non-verbal communication (eye contact, facial expressions, pointing and other gestures), verbal communication (delayed first words, unusual prosody, immediate echolalia and/or stereotyped or idiosyncratic use of words and phrases), reduced shared enjoyment and social smiling; absent or unusual social overtures; inappropriate or odd social responses and reduced conversational reciprocity, severe impairments in all aspects of peer relationships, reduced imaginative play, restricted interests and repetitive behaviours, including unusual preoccupations and compulsions/rituals. Two had received a prior independent clinical autism diagnosis and the third had autism confirmed on clinical examination in this study; two were in specialist educational provision.

Three children (5.6 %) meeting *PDD*-*NOS criteria* (one boy, two girls) had a pattern characteristic of familial idiopathic ASD on the ADI-R and ADOS assessments and all showed evidence of repetitive, restricted and stereotyped interests and behaviours. ADOS-2 total scores were lower than in the autism group, but all above ASD threshold. They initiated social interaction and showed desire to interact and converse with an adult, but their overtures, responses and reciprocity were still atypical. They commonly wanted to make friends and play with other children, but lacked the social insight and flexibility. Parents reported early difficulties with non-verbal communication but ADOS observation highlighted fewer difficulties with pointing, gestures, and facial expressions by middle childhood, their spoken language showed fewer idiosyncrasies, although they often used stereotyped and repetitive speech and socially inappropriate comments/questions. They demonstrated restricted interests and repetitive behaviours, but did not show the very unusual preoccupations or compulsions/rituals seen in the children with autism. On separate clinical review (JG), one was assessed as full ASD, one with partial ASD spectrum features, one with empathy impairment; none were in specialist school provision.

The ten cases (18.5 %) showing ‘*Broad ASD*’ (eight boys, two girls) mainly met ASD criteria on the parent-report ADI, but had lower ADOS-2 scores, with 3/9 meeting ADOS-2 ASD criteria and 4/9 showing significant restricted repetitive behaviours (RRB) on ADOS2. They showed similar autistic-like impairments in early childhood on parent report, involving difficulties in non-verbal communication, socio-emotional reciprocity like offering comfort to others, and imagination. Like the children with ASD they often initiated interaction with others and showed a desire to make friends and play with other children but did not have the skills, often trying to control and dominate interactions, and befriending adults, younger children or children with special needs. On ADOS assessment they were less impaired than the children with ASD, but had reduced reciprocity with attempts to dominate conversation or play and a lack of social insight, with inappropriate questions or embarrassing comments. There were some unusual sensory interests but no language atypicality and manneristic behaviour. Separate clinical assessment by the lead author in 4/9 of these cases diagnosed partial ASD traits in one case and disinhibited attachment disorder or social communication/empathy impairment in others. Case vignettes illustrating each group are included in online supplementary information (S1).

### Language and Communication

Screen positive cases showed reduced overall pragmatic functioning (General Communication Composite on CCC2) compared to screen negative cases (Table 2); but no difference in structural language (CELF Word Classes or Sentence Recall). The data gives some suggestion that the CCC2 social interaction deviance composite, and context, nonverbal and social subscales might be more impaired in the ASD related groups, but the numbers here are too small for strong inferences.

### Other Psychopathology

The screen positive group showed high proportions of emotional disorder and ADHD compared to the screen negative group (Table 2), with no significant difference in externalising problems. Within the screen positive group, there were no relative differences rates of co-morbid conditions identified across autism, PDDNOS or Broad Autism groups. Notably, co-morbidity rates did not differ between screen positive cases that were not diagnosed ASD or Broad ASD (false screen positives), and those who were diagnosed (true positives); suggesting that the in depth phenotyping was specific for ASD and not confounded by other disorder. Two thirds of cases positive for ASD or Broad ASD also met criteria for separately assessed Disinhibited Attachment Disorder (DAD; Kay et al. submitted), but one third showed no such co-occurrence.

### Presence of Aetiological Risk

Risk exposure identified from adoptive parental interview is summarised in Table 3.

**Table 3:** Information related to aetiology (screen positive cohort, n = 21)

	Autism N = 3 (%)	PDD-NOS N = 3 (%)	Broad ASD N = 10 (%)	Non-ASD N = 5 (%)
*Developmental disorder in birth family*				
Autism	2 (67)	0	0	0
Learning difficulties	3 (100)	2 (67)	4 (40)	2 (40)
Mental health difficulties	2 (67)	3 (100)	7 (70)	5 (100)
*Pre*-*natal exposure*				
Pre-natal alcohol exposure (confirmed/suspected)	1 (33)	1 (33)	5 (50)	4 (80)
Foetal Alcohol Spectrum Disorder^b^	1 (33)	0	1 (10)	1 (20)
Pre-natal drug exposure (confirmed/suspected)	0	0	4 (40)	3 (60)
No of Minor Physical Anomalies/subject	3.67 (1.53)	3.00 (1.73)	2.33 (2.40)^a^	3.00 (2.55)
Head circumference >91st percentile	1 (33)	0	0	1 (20)
Head circumference <9th percentile	1 (33)	2 (67)	4 (40)	1(20)
*Post*-*natal exposure*				
Removed at birth	2 (67)	0	4 (40)	1(20)
Neglect in birth family	1 (33)	3 (100)	7 (70)	4 (80)
Physical abuse in birth family	0	2 (67)	4 (40)	2 (40)
Age became looked after, in months	2 at birth	15.33 (10.69)	7.30 (9.81)	15.80 (12)
M (SD)	1 at 2 m			
Number of foster placements/child	2 = 1 placement	3.67 (2.89)	2.60 (1.51)	3.40 (2.2)
M (SD)	1 = 2 placement			

^a^Missing data for one participant

^b^Hoyme criteria (see text)

A birth family history of autism was specific to the autism category and learning difficulty and mental health problems were also highly represented. There was little suggestion that family history differed significantly across other groups.

Two children who met ASD criteria were suspected or confirmed to have had prenatal alcohol exposure. One of these children met criteria for FASD (thin upper lip, smooth philtrum, stature and head circumference less than 10th percentile). Of the ten children showing partial ASD features, five were suspected/confirmed to have been exposed to alcohol and/or drugs prenatally and one of these met FASD criteria. No children had pre-existing clinical diagnoses of FASD. Minor physical anomalies were detected with a mean of 2.8 (SD 2.14; range 1–7) per case. MPA total was linked to a history of drug/alcohol exposure in pregnancy (exposed mean MPA/case = 3.42 (*SD* = 2.43, *n* = 12); non-exposed mean MPA/case = 1.88 (*SD* = 1.25, *n* = 8); and there was a trend towards increased MPA in the autism group, but the numbers are small. In relation to extremes of head circumference, small head circumference (<9th percentile) is strikingly prevalent, particularly in the PDD-NOS group, whereas large head circumference (>91st percentile) is overall less striking although seen in a proportion of autism cases; however numbers are again too small for confident inferences.

Of the three cases of ‘autism’, two were removed into care at birth implying no postnatal risk exposure and one early at two months after neglect. A history of neglect and physical abuse is reported across all other groups and rather marked in the PDD-NOS group. Length of post-natal risk exposure as indexed by age when first taken into care does not show any distinction between PDD-NOS, Broad ASD and non-ASD groups.

## Discussion

This two phase ascertainment study of children in adoption after early disrupted care, neglect or maltreatment used standardised measures and demonstrated a strikingly high incidence of ASD phenotype in 11 %, with a further 18.5 % showing partial features. The children showing ASD are not just presenting with impairment of social reciprocity and empathy, they show the full range of characteristic DSM5 ASD symptoms including repetitive behaviours, unusual interests and stereotyped behaviours (although generally a lack of atypical early language). The pattern of findings on phenotypic ascertainment, confirmed by clinical assessment, suggest a *core group of three cases* with a family history of autism, learning disability or mental illness, who were removed at or very soon after birth and thus exposed to little or no postnatal environmental family risk. These cases seem characteristic of a familial idiopathic autism or other pre-natal risk, which has clustered in families whose parents have proved unable to manage a child who has come into early care. A further group of *three PDD*-*NOS cases* do not show birth-family history of autism but do report birth family learning difficulty and/or mental health problems with reported exposure to pre-and/or post-natal adversity; notably, high levels of parental mental illness, small head circumference, post-natal neglect or physical abuse. All PDD-NOS cases showed a full range of ASD symptomatology but in less severe form; all but one showed a full or partial ASD syndrome on clinical assessment. Of the *ten cases showing ‘Broad ASD’*, clinical assessment on four reported ASD or partial traits (one of these with comorbid DAD); two showed DAD without ASD traits; three showed ADHD (one comorbid with ASD); and others a mixture of non-specific attachment and sensory processing features.

The approach to in-person ASD phenotype ascertainment used in this study allows comparison with results from the equivalent assessment approach taken in the Romanian adoptees study (Rutter et al. 1999). The ASD syndrome profile shows a similar range in both studies, notably including repetitive, stereotyped behaviours and restricted interests as well as social impairment; and similar features such as the equal gender ratio and lack of atypical head size. However, as noted in the introduction, the context of the two cohorts differs. Whereas the Romanian adoptees the focus of difficulty was on the post-natal social deprivation also seen in our sample, here we find in addition pre-natal and family risk exposures, which are increasingly typical of children coming into adopted care. Q–A in the ERA cohort showed a unique developmental progress in development that was different to autism, longitudinal follow-up in this study is underway and will be important to understand the developmental course of our findings. Our study is unique to our knowledge in using standardised in-person assessment in this context, but its findings are consistent with other research on children post adoption. Thus in-depth study of 35 families from a recent survey of 390 adoptive parents in England (Selwyn et al. 2014) reported independent autism diagnosis in 11 %; earlier accounts of autistic-like detachment in 26 % of 70 children at entry into care (Dimigen et al. 1999) and impaired theory of mind in children after maltreatment (Cicchetti et al. 2003; Kay and Green 2015).

In addition to ASD, we report high levels of co-morbidity across all the screen-positive group (see Table 2) and this is an important feature of this clinical area. However, the rates of comorbidity do not vary between autism, PDDNOS or Broad Autism categories, and suggests that our CPEA-based research diagnoses of autism are specific and not misdiagnoses confounded by concurrent other disorder. Between screen positive and screen-negative cases levels of emotional disorder and ADHD do differ; this may reflect common-rater biases operating in parent-report based measures and emphasizes the value of in-person assessment of ASD.

Regarding limitations: we consider the findings in this sample as striking and important in their own right but cannot be sure yet quite how typical they are of UK adoption as a whole. The families self-referred in response to an invitation worded to be non-specific without mention of autism or any other specific hypothesis. The majority of children referred had suffered significant neglect and maltreatment and in this they are very typical of the general pattern now current within UK adoption where 72 % of children with adoption orders first become looked after due to maltreatment at an average age of 1.2 years, and roughly two thirds are adopted before the age of 4 years (Selwyn et al. 2014). We have no evidence therefore of significant sampling bias affecting our results, although this cannot be excluded. Confirmatory survey data is planned to establish prevalence rates applicable to the wider population of UK adopted children. The method of ascertainment of prior risk exposures through adoptive parent report was consistent across the study, but it is clearly possible that, despite our best efforts, the developmental interview data under-represents or biases the actual extent of risk exposure in these children. The practicality of accessing confirmatory medical or social care records was very variable, due to the lack of contact with social services and birth families, the need for birth family consent for records access and the time elapsed since adoption; it was considered that using the records in some cases but not others would be misleading and result itself in differential assessment biases. In the current environment of UK adoption (typical of other high income countries), there was no opportunity to design a comparison group of children adopted without early adversity. For instance, the group of children adopted at birth (thus avoiding postnatal adversity) are almost universally adopted because of overwhelming concern about familial or prenatal risk exposures - and our data reflects this. Detailed phenotyping was untaken on screen positive cases only; resources precluded assessing screen negative cases also. However, our study was focused on a conservative ascertainment of ASD rather than testing the specificity of the screen measure, and we think the risk of not having identified true ASD cases in the sample is low. Our identification of ASD using CPEA criteria was chosen as the best available systematic way to integrate the ADI and ADOS research assessments into a classification scheme; it does not replicate a full clinical ascertainment but has shown added value in presenting a standardized quantifiable approach to classification which can avoid the vagaries of clinical diagnostic usage (Lord et al. 2012a, b).

These results are suggestive but do not yet allow specific inferences for developmental theory. Our data suggests that there are likely be a variety of convergent familial, pre-and postnatal risks responsible for the high rates of ASD symptoms seen here but precisely which and in how they are interacting in most cases cannot be clearly determined from this retrospective study. The majority of children had significant exposure to severe neglect and maltreatment in their birth families but there is no simple evidence of a “dosage” effect of length of postnatal exposure in relation to later outcome, as was found within the ERA study (Kumsta et al. 2010). Further hypothesis-driven research designs are indicated, ideally including prospective ascertainment of at risk groups in the prenatal period.

The clinical implications of these results are however immediate and significant, since identification of ASD impairments has specific implications for family understanding, style of intervention, and educational planning. Anecdotally, adopting families across our study often reported feeling isolated, with a lack of appropriate services and unsure as to whether to ascribe their child’s difficulties to attachment or emotional disorder, stubbornness, rejection, or developmental difficulty; reports that echo recent large-scale formal surveys of adopting parents (Pennington 2012; Selwyn et al. 2014). Services themselves are faced with adjustment to the increasing complexity in risk background of children coming into adoption; a common response to a child’s difficulty in engagement with an adoptive placement or destructive or disruptive behaviour has been to ascribe a psychological model of attachment disruption, emotional inhibition or post-traumatic disturbance. Clearly all of these phenomena may be significant in children with such pasts, but the presence of neurodevelopmental disorder such as ASD is an important alternative explanation for the child’s difficulties, with different implications. Informed assessment that can characterise developmental disorder as well as psychological disturbance in these complex cases is essential and will lead to much more efficient and targeted management. It is also possible that these social impairments, if persistent, could contribute to the very poor functional outcomes sometimes reported after adoption; the extent of functional impairment associated with quasi autism or attachment disorder in the ERA cohort emphasise this point. Understanding of the neurodevelopmental consequences of the combination of early biological and environmental risk exposure should result in a paradigm shift; opening up new ways of understanding and managing the problems that often arise now for children and families post-adoption.

## Electronic supplementary material

Supplementary material 1 (DOCX 18 kb)

## References

[CR101] Aebi M, Kuhn C, Metzke CW, Stringaris A, Goodman R, Steinhausen HC (2012). The use of the development and well-being assessment (DAWBA) in clinical practice: A randomized trial. European Child and Adolescent Psychiatry.

[CR102] Alyahari A, Goodman R (2006). Validation of the Arabic Strengths and Difficulties Questionnaire and the Development and Well-Being Assessment. East Mediterranean Health Journal.

[CR1] Bishop D (2001). The Children’s Communication Checklist, version 2 (CCC-2).

[CR2] Botting N, Conti-Ramsden G (2008). The role of language, social cognition, and social skill in the functional social outcomes of young adolescents with and without a history of SLI. British Journal of Developmental Psychology.

[CR3] Bromley R, Mawer GE, Briggs M (2013). The prevalence of neurodevelopmental disorders in children prenatally exposed to antiepileptic drugs. Journal of Neurology, Neurosurgery and Psychiatry.

[CR4] Cicchetti D, Rogosch FA, Maughan A, Toth SL, Bruce J (2003). False belief understanding in maltreated children. Development and Psychopathology.

[CR105] Colvert E, Tick B, McEwen F, Stewart C, Curran SR, Woodhouse E (2015). Heritability of autism spectrum disorder in a UK population-based twin sample. Journal of the American Medical Association Psychiatry.

[CR5] Dimigen G, Del Priore C, Butler S (1999). Psychiatric disorder among children at time of entering local authority care: Questionnaire survey. British Medical Journal.

[CR6] Elsabbagh M, Divan G, Koh YJ (2012). Global Prevalence of Autism and Other Pervasive Developmental Disorders. Autism Research.

[CR7] Ford T, Vostanis P, Meltzer H, Goodman R (2007). Psychiatric disorder among British children looked after by local authorities: Comparison with children living in private households. British Journal of Psychiatry.

[CR8] Goodman R, Ford T, Richards H (2000). The Development and Well-Being Assessment: Description and initial validation of an integrated assessement of child and adolescent psychopathology. Journal of Child Psychology and Psychiatry.

[CR9] Hoyme HE, May PA, Kalberg WO (2005). A practical clinical approach to diagnosis of fetal alcohol spectrum disorders: Clarification of the 1996 institute of medicine criteria. Pediatrics.

[CR10] Jenkins EA (2006). A study of minor physical anomalies in twin pairs ages 5–12 years: A predicator of behavioural variation.

[CR11] Kay CL, Green JM (2015). Social cognitive deficits and biases in maltreated adolescents in UK out-of-home care: Relation to disinhibited attachment disorder and psychopathology. Development and Psychopathology.

[CR12] Kreppner J, Kumsta R, Rutter M (2010). Developmental course of deprivation-specific psychological patterns: Early manifestations, persistence to age 15, and clinical features. Monographs of the Society for Research in Child Development.

[CR13] Kumsta R, Kreppner J, Rutter M (2010). Deprivation-specific psychological patterns. Monographs of the Society for Research in Child Development.

[CR14] Lainhart JE, Bigler ED, Bocian M (2006). Head circumference and height in autism: A study by the collaborative program of excellence in autism. American Journal of Medical Genetics.

[CR15] Le Couteur A, Lord C, Rutter M (2003). The autism diagnostic interview - Revised (ADI-R).

[CR16] Lord C, DiLavore P, Risi S (2012). Autism Diagnostic Observation Schedule - 2nd edition (ADOS).

[CR17] Lord C, Petkova E, Hus V (2012). A multisite study of the clinical diagnosis of different autism spectrum disorders. Archives of General Psychiatry.

[CR100] Meltzer H, Gatward R, Goodman R, Ford T (2000). Mental health of children and adolescents in Great Britain.

[CR18] Miles J, Takahashi TN, Haber A (2003). Autism Families with a High Incidence of Alcoholism. Journal of Autism and Developmental Disorders.

[CR19] O’Connor TG, Monk C, Fitelson EM (2014). Practitioner Review: Maternal mood in pregnancy and child development - Implications for child psychology and psychiatry. Journal of Child Psychology and Psychiatry.

[CR103] Pennington. (2012). It takes a village to raise a child. Retrieved from http://www.adoptionuk.org/sites/default/files/documents/Ittakesavillagetoraiseachild-Report-June12.pdf.

[CR20] Rai D, Lee BK, Dalman C (2013). Parental depression, maternal antidepressant use during pregnancy, and risk of autism spectrum disorders: Population based case-control study. British Medical Journal.

[CR21] Rees CA, Selwyn J (2009). Non-infant adoption from care: Lessons for safeguarding children. Child: Care, Health and Development.

[CR22] Ronald A, Pennell CE, Whitehouse AJO (2011). Prenatal maternal stress associated with ADHD and autistic traits in early childhood. Frontiers in Psychology.

[CR23] Rutter M, Andersen-Wood L, Beckett C (1999). Quasi-autistic patterns following severe early global privation. Journal of Child Psychology and Psychiatry.

[CR24] Rutter M, Kreppner J, Croft C (2007). Early adolescent outcomes of institutionally deprived and non-deprived adoptees. III. Quasi-autism. Journal of Child Psychology and Psychiatry.

[CR25] Sadiq FA, Slator L, Skuse D (2012). Social use of language in children with reactive attachment disorder and autism spectrum disorders. European Child and Adolescent Psychiatry.

[CR26] Selwyn J, Wijedasa D, Meakings S (2014). Beyond the adoption order: Adoption disruption and families in crisis.

