# Supplementary material for: Autism Spectrum Disorder in Children Adopted After Early Care Breakdown
Source: J Autism Dev Disord. 2016 Jan 6;46:1392–402. doi: 10.1007/s10803-015-2680-6 (PMC4786596; doi:10.1007/s10803-015-2680-6)
Supplement: Supplementary file 1 — Supplementary material 1 (DOCX 18 kb) [file 10803_2015_2680_MOESM1_ESM.docx]

**Supplementary Information**

**Illustrative Case Vignettes**

**Case 1**

Jack had a preterm birth at 34 weeks. There was no definitely known prenatal environmental exposure to alcohol or drugs during pregnancy. At birth he spent 5 days in special care with a low APGAR score. He had conductive hearing loss as an infant and grommets at age 2.

Birth mother had learning difficulties with a reported IQ of <70. She was reported to be socially unusual and did not engage with services. Birth father was said to be socially reticent and experienced anxiety and depression, although had no known learning disability. There was conflict and violence between the parents. Jack has one full sister and two half-brothers from the birth family. One half-brother has a diagnosis of autism and learning difficulties; the other also has developmental difficulties.

Jack was taken into care at 2 years following severe neglect and emotional abuse, ongoing since birth. The house was in a completely chaotic state and dirty with animal excrement. Jack was left for long periods of time without being fed or changed, sometimes in the care of his sister who was two years older than him, and was often found strapped into a car seat. His emotional needs were not met. It is suspected that his older sister was sexually abused.

Jack was in a temporary placement for 6-8 weeks and then placed with a second foster home for 18 weeks. He was adopted by his current adoptive parents at 3 years 9 months.

Upon adoptive placement, Jack had a diagnosis of developmental delay. He showed brachycephaly (a flat back of the head often associated with prolonged lying supine). He was highly agitated at any restraint. He was virtually without language. He made early and swift catch up in language within adoptive care, followed by progress in cognitive skills and motor skills. He is now thought to be in the normal IQ range but has continuing social difficulties.

At assessment Jack was 7 years 4 months. He was attending a mainstream primary school and had an educational statement of special needs with 20 hours of one-to-one support. He was said to be functioning at the first centile in non-verbal reasoning. His hearing was now within normal limits.

Jack scored above autism cut-offs on the ADI-R interview, carried out with his adoptive mother and father, and on the ADOS. Both the interview and play-based observations indicated considerable social communication difficulties, particularly with using and understanding non-verbal communication (eye contact, gestures, facial expressions), atypical features of speech, engaging in flexible and reciprocal interactions with others, picking up on social cues to guide interactions and behaviours, and with imagination and flexibility of thought and behaviour.

Jack showed some motor immaturity with a palmer grip in his left hand and drawing characteristic of a younger child. Jack looked mildly dysmorphic. He had a broad nasal bridge, relatively flat philtrum; and a relatively large head with brachycephaly. Ears looked normal.

Clinical assessment (JG) Developmental Coordination Disorder and Autistic Spectrum Disorder.

**Case 2**

Matthew was born at full term. His birth was said to be traumatic, resulting in an emergency C-section. Matthew was born with breathing difficulties and a cleft palate, and received emergency treatment for two weeks.

Birth mother had suspected mental health problems and low cognitive functioning. She drank heavily although it is unknown the extent to which she drank during pregnancy. Birth father had depression, including stays in a psychiatric hospital, and also low cognitive functioning. He also drank heavily. Matthew has three birth siblings.

Matthew’s birth family had been known to social services for a long time due to their poor functioning and chaotic lifestyle, and substandard living conditions. The children witnessed domestic violence. Matthew was known to services since birth. The children missed health appointments and had poor attendance at nursery. Family support was rejected. Matthew was taken into care at 21 months because of assessed parental neglect and possible physical abuse (sibling with unexplained bruising). He was placed in an emergency foster placement and then with foster carers for 18 months. He was placed within an adoptive family, alongside his birth brother, at 3 years 3 months.

When seen for research assessments, at 10 years 2 months, Matthew was attending a mainstream primary school, with a statement of SEN and receiving 20 hours a week additional support. He had received involvement from Child and Adolescent Mental Health services who had identified complex social and emotional difficulties and attachment difficulties. Two of Matthew’s birth siblings showed social and attentional difficulties.

Upon assessment Matthew was a charming and engaging boy who is very communicative and socially approaching. He showed a number of distinct idiosyncrasies in communication style at interview. His eye gaze was non-fluent and often relatively fixed. He used unusual prosody (the timbre and pitch regulation) and concrete and stereotyped language. The linkage of verbal to non-verbal communications was often mistimed. Matthew showed some mild manneristic movements. He showed a vivid attention to detail and mild preoccupation with particular topics.

Matthew was mildly dysmorphic with thin upper lip, clinodactyly of fifth fingers, and slightly unusual ears.

Matthew’s scores on the ADI-R, carried out with his adoptive mother, were well above the autism threshold, and his scores on the ADOS were above threshold for reciprocal social interaction and borderline for communication. He showed some difficulty with ‘theory of mind’. Clinical assessment (JG) was of Asperger Syndrome.

**Case 3**

Brandon was brought into care at the age of 2 1/2 years for reasons of emotional abuse and neglect. His birth mother was 16 years old at Brandon's birth and was very late receiving pre-natal care. The pregnancy went uneventfully as far as is known with no known drug or alcohol abuse. The birth was by emergency Caesarean section at term but there was no record for foetal trauma or distress.

Birth mother had herself grown up in a chaotic family and subject to (probably commercial) sexual abuse with a neighbour mediated by mother. During her pregnancy her own mother had an affair with the child's father. Birth father noted to be of average to good intelligence with no family history of mental health or developmental disorder. Brandon has one younger birth half-sister.

When Brandon was six weeks old birth mother was thrown out of the house by her own mother and there then followed the period of gross instability with 20 house moves in 18 months, criminal activity, unstable parenting, domestic violence and conflict, likely postnatal depression, deprivation of food (hospitalized with malnutrition), gross neglect (strapped in pram for long periods, left for days in the care of 7 year olds), and unpredictable behaviour from mother. There was no evidence of significant physical abuse. At reception into foster care aged 2 1/2 it was noted that Brandon's speech and language development was very poor and that he would “howl for food”. It took him a considerable time to begin to ask for what he needed and food remained a significant issue in his behavioural reactions.

When removed from the care of his birth family Brandon was placed in emergency foster care for 3 days. He then returned home for two months before being taken permanently into care at 2 ½. He lived within an excellent foster care environment before being placed with his adoptive family, along with his half-sister, at 3 ½ years.

Brandon was assessed at 12 years, 2 months. He was attending a mainstream secondary school with no additional learning or behavioural support, apart from some additional pastoral care. He had no identified special needs or diagnoses. He scored above cut-offs for autism on the ADI-R interview, using information from his adoptive parents based mainly on when he was aged 4-5 years. He scored below cut-off for ASD on the ADOS.

Clinically Brandon presented as a polite pleasant boy who conformed to what was asked of him and engaged well in conversation. He showed no significant abnormality of social communication either verbally or nonverbally in the interview situation, apart from a subdued range of expression and gesture.

Diagnostically (JG) Brandon showed as sub-threshold for ASD. He showed indications of a disinhibited attachment disorder and avoidant emotional/attachment style. He showed poor attention in relation to initiation, persistence, distractibility and disinhibition. In addition he showed evidence of social cognition impairments and an empathy impairment.
